# Prognostic Role of Inflammatory and Nutritional Indices in NSCLC Patients Treated with Immune Checkpoint Inhibitors: Retrospective, Multicenter, Turkish Oncology Group Study of Overall and Elderly Populations

**DOI:** 10.3390/medicina61071160

**Published:** 2025-06-26

**Authors:** Ozkan Alan, Tugba Akın Telli, Sinem Akbas, Selver Isik, Eyyüb Çavdar, Kubilay Karaboyun, Aysegül Merc Cetinkaya, Ferhat Ekinci, Atike Pınar Erdoğan, Mahmut Büyükşimsek, Muhammed Muhittin Er, Melek Karakurt Eryilmaz, Taliha Güçlü Kantar, Gamze Gököz Doğu, Teoman Sakalar, Ertuğrul Bayram, Ali Inal, Fatma Akdağ Kahvecioğlu, İlhan Hacibekiroğlu, Fatih Selçukbiricik, Ali Murat Tatli, Perran Fulden Yumuk

**Affiliations:** 1 Division of Medical Oncology, Department of Internal Medicine, Cerrahpaşa Faculty of Medicine, Istanbul University-Cerrahpaşa, Istanbul 34098, Türkiye; 2 Division of Medical Oncology, Department of Internal Medicine, Demiroglu Bilim University, Istanbul 34394, Türkiye; tugbaakintelli@gmail.com; 3 Division of Medical Oncology, Department of Internal Medicine, Koc University School of Medicine, Istanbul 34450, Türkiye; sakbas@kuh.ku.edu.tr (S.A.); fselcukbiricik@kuh.ku.edu.tr (F.S.); fuldenyumuk@yahoo.com (P.F.Y.); 4 Division of Medical Oncology, Department of Internal Medicine, Marmara University School of Medicine, Istanbul 34854, Türkiye; selverr83@gmail.com; 5 Division of Medical Oncology, Department of Internal Medicine, Tekirdağ Namık Kemal University, Tekirdag 59030, Türkiye; eyyupcavdar@hotmail.com (E.Ç.); kubilaykaraboyun@gmail.com (K.K.); 6 Division of Medical Oncology, Department of Internal Medicine, Akdeniz University, Antalya 07058, Türkiye; aysegul_merc@hotmail.com; 7 Division of Medical Oncology, Department of Internal Medicine, Celal Bayar University, Manisa 45300, Türkiye; drferhatekinci@hotmail.com (F.E.); atike.erdogan@cbu.edu.tr (A.P.E.); 8 Division of Medical Oncology, Department of Internal Medicine, Adana City Hospital, Adana 01230, Türkiye; mahmutbuyuksimsek@gmail.com; 9 Division of Medical Oncology, Department of Internal Medicine, Necmettin Erbakan University School of Medicine, Konya 42080, Türkiye; muhiddiner09@gmail.com (M.M.E.); drangelkarakurt@hotmail.com (M.K.E.); 10 Division of Medical Oncology, Department of Internal Medicine, Faculty of Medicine, Pamukkale University, Denizli 20160, Türkiye; talihaguclu@hotmail.com (T.G.K.); ggd2882@gmail.com (G.G.D.); 11 Division of Medical Oncology, Department of Internal Medicine, Kahramanmaraş Necip Fazıl City Hospital, Kahramanmaras 46050, Türkiye; drteomansakalar@gmail.com; 12 Division of Medical Oncology, Department of Internal Medicine, Faculty of Medicine, Cukurova University, Adana 01330, Türkiye; ertugrulbayram84@gmail.com; 13 Division of Medical Oncology, Department of Internal Medicine, Mersin City Hospital, Mersin 33330, Türkiye; dr.ainal@gmail.com; 14 Division of Medical Oncology, Department of Internal Medicine, Faculty of Medicine, Sakarya University, Sakarya 54290, Türkiye; fatmaakdag_87@hotmail.com (F.A.K.); ilhanhbo@hotmail.com (İ.H.); 15 Division of Medical Oncology, Memorial Antalya Hospital, Antalya 07025, Türkiye; alimurattat@hotmail.com

**Keywords:** non-small-cell lung cancer (NSCLC), prognostic nutritional index (PNI), geriatric nutritional risk index (GNRI), immune checkpoint inhibitors, nivolumab, inflammation-based indices

## Abstract

*Background and Objectives:* Despite advances in immunotherapy, predicting survival outcomes in patients with non-small-cell lung cancer (NSCLC) remains challenging. Inflammatory and nutritional indices such as the Prognostic Nutritional Index (PNI), Geriatric Nutritional Risk Index (GNRI), Neutrophil-to-Lymphocyte Ratio (NLR), Platelet-to-Lymphocyte Ratio (PLR), and Inflammatory Burden Index (IBI) have emerged as promising prognostic markers associated with overall survival (OS) in NSCLC patients. *Materials and Methods:* We retrospectively analyzed a total of 196 NSCLC patients treated with second-line nivolumab across multiple centers in Turkey. Of these, 101 patients aged ≥ 65 years were included in the elderly subgroup analysis. PNI, GNRI (in patients aged ≥ 65), and inflammation-based indices were calculated using pre-treatment laboratory values. ROC analysis determined optimal cut-off values. The Kaplan–Meier method and Cox proportional hazards models were used for survival analysis. *Results:* Median overall survival (OS) was 12.9 months in the full cohort and 12.1 months in patients aged ≥ 65. In univariate analysis, ECOG performance status (0–1), lower NLR (<3.3), lower PLR (<196.8), higher PNI (≥45.2), and higher GNRI (≥98.0) were significantly associated with longer OS. However, in the multivariate analysis adjusted for ECOG PS, NLR, PLR, and GNRI, only PNI remained an independent prognostic factor for OS in both the overall cohort [HR: 0.49, 95% CI: 0.26–0.92; *p* = 0.02] and elderly patients [HR: 0.45, 95% CI: 0.24–0.84; *p* = 0.01]. PNI is an independent prognostic biomarker for OS in NSCLC patients treated with immune checkpoint inhibitors. *Conclusions:* These findings support incorporating simple, cost-effective nutritional indices into clinical decision-making, particularly in elderly patients with NSCLC.

## 1. Introduction

Non-small-cell lung cancer (NSCLC) continues to be one of the most prevalent causes of cancer-related deaths globally, despite significant progress in treatment modalities. Worldwide, lung cancer affects approximately 2.1 million individuals and accounts for an estimated 1.8 million deaths annually [1]. Among recent therapeutic advancements, immune checkpoint inhibitors—particularly those that inhibit the PD-1/PD-L1 pathway, such as nivolumab—have demonstrated improved survival outcomes in patients with advanced NSCLC who have experienced disease progression following platinum-based chemotherapy regimens [2]. Nivolumab was approved by the FDA in 2015 for the treatment of advanced NSCLC in the second-line setting. Nonetheless, the variability in patient responses to immunotherapy underscores the urgent need for practical and reliable prognostic markers that have been validated across diverse populations in order to improve patient selection and guide therapeutic decision-making.

In recent years, systemic inflammatory markers and indicators of nutritional status have gained prominence as prognostic tools in oncology. Easily obtainable from routine blood tests, indices such as the Prognostic Nutritional Index (PNI), Geriatric Nutritional Risk Index (GNRI), Neutrophil-to-Lymphocyte Ratio (NLR), Platelet-to-Lymphocyte Ratio (PLR), and Inflammatory Burden Index (IBI) have been explored for their prognostic relevance across various cancer types, including NSCLC [3,4,5]. These markers are particularly advantageous due to their cost-effectiveness, non-invasiveness, and potential applicability in elderly or comorbidities patient populations.

In this multicenter, retrospective study, we aimed to evaluate the prognostic value of nutrition- and inflammation-based tools in patients with NSCLC treated with second-line nivolumab.

## 2. Materials and Methods

### 2.1. Patients and Methods

#### Patients

We retrospectively collected demographic, clinicopathologic, and laboratory data from 196 NSCLC patients treated with second-line immunotherapy (Nivolumab) between January 2020 and December 2023 in a multicenter study in Turkey. The inclusion criteria were as follows: histologically or cytologically confirmed diagnosis of NSCLC, disease progression after first-line treatment, treatment with single-agent immunotherapy, and the availability of complete medical records including laboratory results, radiologic response, and survival data. The exclusion criteria included patients with incomplete radiological assessment of treatment response, insufficient clinical documentation, the presence of known driver mutations, active infections, autoimmune diseases, concurrent malignancies, or age under 18 years.

All patients included in this study received platinum-based chemotherapy as first-line treatment. None of the patients were treated with first-line immunotherapy as it was not reimbursed in our country during the study period.

### 2.2. Prognostic Nutritional Index (PNI) and Geriatric Nutritional Index (GNRI)

PNI and GNRI were calculated using lymphocyte count, serum albumin levels, and current body weight and height obtained from routine blood tests performed prior to the first dose of nivolumab. GNRI was calculated only for patients aged 65 years and older.

PNI was calculated as follows: [(10 × serum albumin (g/dL)) + (0.005 × total lymphocyte count (10^3^/µL))].

GNRI was calculated as follows: [1.489 × albumin (g/dL)] + [41.7 × (weight/Ideal Body Weight)].

Ideal body weight (IBW) was calculated using the modified Devine formula: 50.0 + 0.91 × (height in cm − 152.4) for males and 45.5 + 0.91 × (height in cm − 152.4) for females [6].

#### Inflammatory-Based Indices

Neutrophil, lymphocyte, platelet, and monocyte counts, along with CRP levels, were obtained from routine blood tests performed within three days prior to the initiation of nivolumab. These values were used to calculate inflammatory-based indices. The following indices were calculated:

Neutrophil–Lymphocyte Ratio (NLR): Neutrophil count/lymphocyte count
Platelet–Lymphocyte Ratio (PLR): Platelet count/lymphocyte count
Inflammatory Burden Index (IBI): CRP (mg/L) × neutrophil-to-lymphocyte ratio

### 2.3. Statistical Analysis

Progression-free survival (PFS) was defined as the time from the date of nivolumab initiation to the first radiologically or pathologically confirmed recurrence of disease, or death from any cause, whichever occurred first. Overall survival time (OS) was defined as the time from the date of nivolumab initiation until death for any reason or the last documented clinical follow-up. Survival analysis was performed using the Kaplan–Meier method. Patients who were lost to follow-up were censored at the time of their last documented contact. Prognostic factors were compared using the log-rank test in univariate analyses. Hazard ratios (HRs) with 95% confidence intervals (CIs) were also calculated. All *p*-values were calculated using two-tailed tests, and a *p*-value of <0.05 was considered statistically significant. Multivariate analysis was carried out using the Cox proportional hazards model to assess the effects of prognostic factors on OS. To determine the optimal cut-off value of indices for predicting overall survival, receiver operating characteristic (ROC) analysis was performed using SPSS version 25 (IBM Corp., Armonk, NY, USA) with the ROC module. All statistical analyses were performed using IBM SPSS Statistics for Windows, version 25.0 (IBM Corp., Armonk, NY, USA)

### 2.4. Optimal Cut-Off Values for Prognostic Markers for Overall Survival

**Figure 1:**
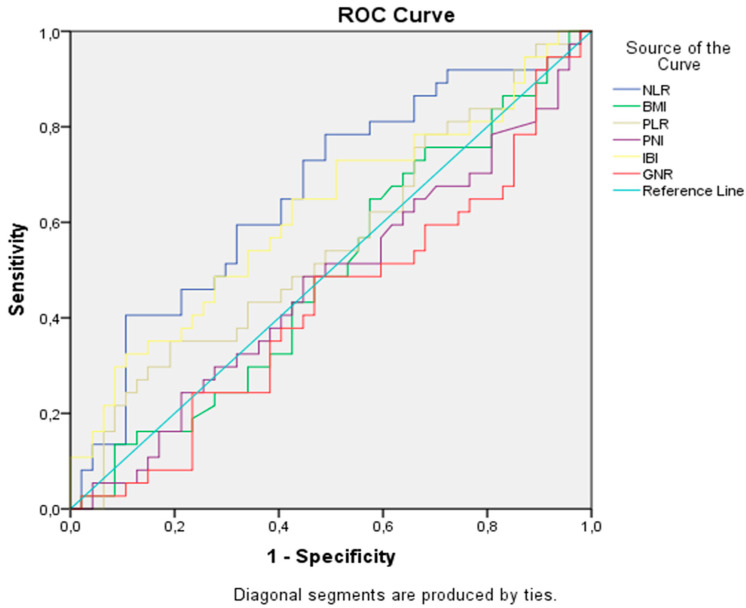
Receiver operating characteristic analysis of prognostic indicators for overall survival.

A significant cut-off value was identified only for NLR. In the ROC analysis, the ideal NLR cut-off value for OS was 3.3 [AUC: 0.66 (0.54–0.78), *p* = 0.01], with a sensitivity of 73% and a specificity of 57% (Figure 1). Median values for PNI, PLR, and IBI were used as the optimal cut-off values. The cut-off value for GNRI was determined based on previous publications and set at 98 [7]. Patients were categorized based on the determined cut-off values.

## 3. Results

### 3.1. Demographic and Clinicopathologic Characteristics of Patients

We collected data from 15 oncology centers across Türkiye and included a total of 196 patients in the study. A total of 167 of 196 were male (85.2%) and the median age was 65 (range 23–88). A total of 51.5% of patients were aged 65 years or older, and 26.5% were active smokers. The most common histological subtype was adenocarcinoma (51.8%). At the time of diagnosis, the clinical stage distribution of the patients was as follows: Stage I in 10 patients (5.1%), Stage II in 35 patients (17.9%), Stage III in 52 patients (26.5%), and Stage IV in 99 patients (50.5%). Regarding the initial treatment approach, 40 patients (20.4%) underwent curative surgical resection, 53 patients (27%) received definitive chemoradiotherapy, and 4 patients (2%) were treated with definitive radiotherapy. PD-L1 expression was assessed via immunohistochemistry (IHC) using tumor tissue samples, and 69 patients (35.2%) had a PD-L1 level of ≥1%, with a median PD-L1 level of 35% (range: 1–95%). Median values for PNI and GNRI were 45.2 (range: 24–60) and 103.8 (range: 64.6–139.31), respectively. Median NLR, PLR, and IBI were calculated as 3.7 (range: 0.9–21.5), 196.8 (range: 26.6–1970), and 60.2 (range: 0.17–236.2), respectively. Baseline demographic and clinicopathologic findings are summarized in Table 1.

### 3.2. Survival Outcomes

**Figure 2:**
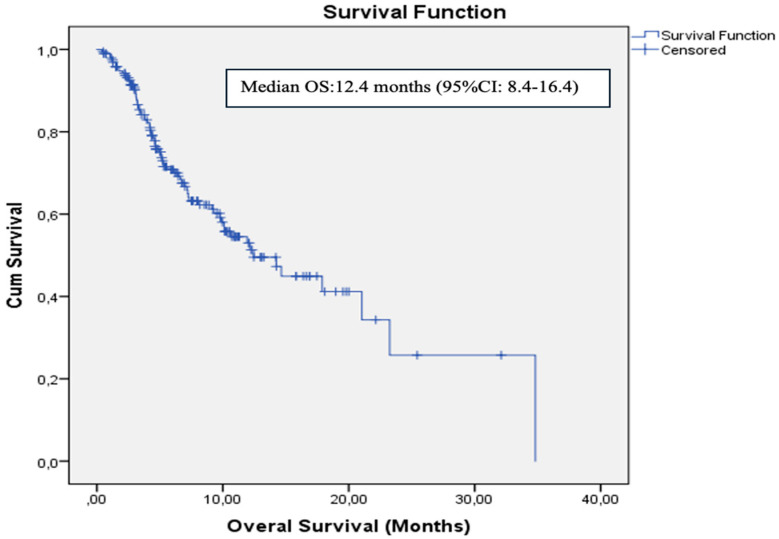
Kaplan–Meier curve for overall survival in the whole cohort.

**Figure 3:**
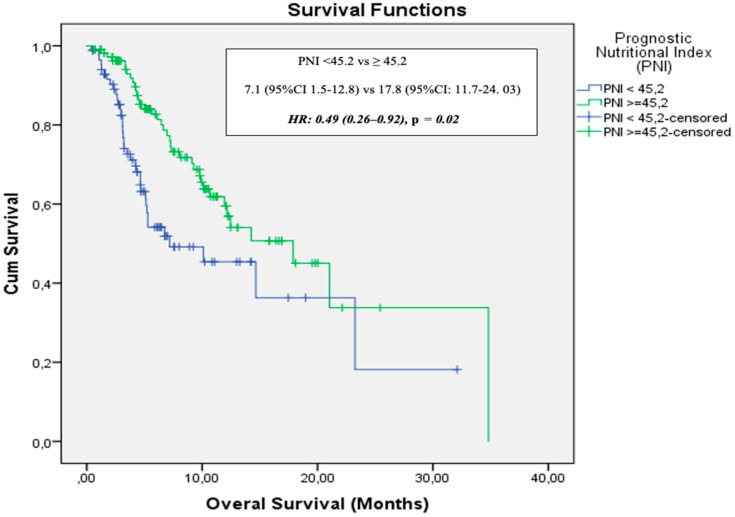
Overall survival stratified by prognostic nutritional index.

The data cutoff date for follow-up was 31 May 2024. The median follow-up duration was 5.3 months (range: 1–34.8 months). During the follow-up period, 134 patients (68.4%) experienced disease progression and 74 patients (37.8%) died. In the entire cohort, median PFS was 4.2 months (95% CI: 3.5–4.8) and median OS was 12.4 months (95% CI: 8.4–16.4). The 12- and 24-month OS rates were 53% and 25%, respectively (Figure 2). ECOG PS, NLR, PLR, PNI, and GNRI were significantly associated with OS in univariate analysis. However, in the multivariate analysis (adjusted for age, ECOG PS, NLR, PLR, PNI, and GNRI), only PNI was identified as an independent prognostic factor for OS [HR: 0.49 (95% CI: 0.26–0.92), *p* = 0.02] (Figure 3). Results of the univariate analysis and OS are presented in Table 2.

**Figure 4:**
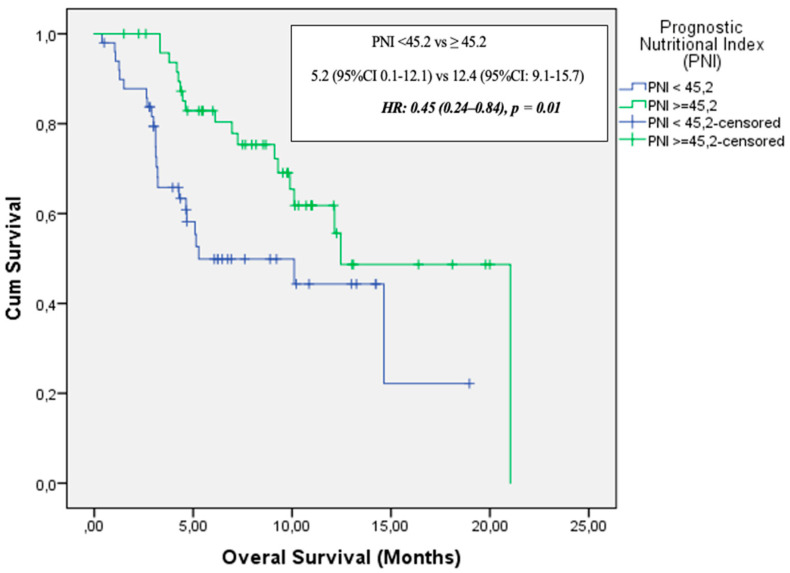
Overall survival by PNI in elderly patients (≥65 Years).

In the elderly cohort (aged 65 years and older), median follow-up duration was 6.1 months (range: 1–21.04 months). During this period, 42 patients (41.6%) died and 67 patients (70%) experienced disease progression. Median PFS was 4.6 months (95% CI: 2.9–6.3), while median OS was 12.1 months (95% CI: 8.7–15.6). In the univariate analysis, ECOG PS, PNI, and GNRI were significantly associated with OS. Multivariate analysis (adjusted for age, ECOG PS, PNI, and GNRI) identified only PNI as an independent prognostic factor for OS [HR: 0.45 (95% CI: 0.24–0.84), *p* = 0.01] (Figure 4). Table 3 presents the univariate analysis results and OS data for patients ≥65 years.

## 4. Discussion

In our study, PNI was identified as an independent prognostic factor for OS in the entire NSCLC cohort, as well as in patients aged 65 years and older. Patients with a high PNI demonstrated significantly improved OS. Although GNI was associated with better OS, it was not an independent prognostic factor.

PNI is an inflammation-based prognostic scoring system derived from two inflammatory markers: lymphocyte count and serum albumin. Initially developed to evaluate the prognostic impact of nutritional and immunological status in malnourished cancer patients undergoing gastrointestinal surgery by Onedera et al., PNI has since been recognized as an independent and valuable prognostic tool for predicting survival in various malignancies, including colorectal and genitourinary cancers and glial tumors [8,9,10,11,12].

**Figure 5:**
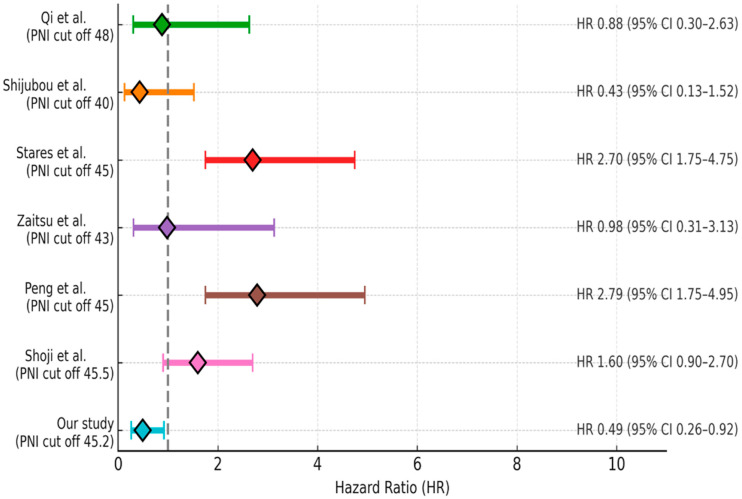
Summary of the current literature on the association between PNI and survival outcomes in lung cancer patients treated with immune checkpoint inhibitors [19,20,21,22,23,24].

Malnutrition and muscle loss are highly prevalent among patients with NSCLC, affecting approximately 70% of cases, which underscores the critical importance of nutritional assessment during anti-cancer treatment [13]. Pretreatment prognostic nutritional indices have been associated with the therapeutic efficacy of various treatment modalities, including targeted therapy, chemotherapy, and radiotherapy in lung cancer patients [14,15]. With respect to immune checkpoint inhibitor (ICI)-based immunotherapy, evidence from a small cohort study of NSCLC patients (*n* = 24) treated with anti-PD-L1 indicated that a higher PNI was linked to a longer time to treatment failure and improved OS [16]. Fang et al. reported that pretreatment PNI served as an independent prognostic marker for OS in a cohort of 223 advanced NSCLC patients receiving anti-PD-1 therapy [17]. In a meta-analysis conducted by Xuebing Yan et al., a total of 1260 patients from 14 studies were evaluated. High PNI level was significantly associated with improved OS (HR = 2.56, 95% CI: 1.86–3.54) and PFS (HR = 1.91, 95% CI: 1.53–2.40) in patients with lung cancer. Subgroup analyses supported these findings, except for PFS in patients receiving anti-PD-1 therapy, where the association was not statistically significant (HR = 1.51, 95% CI: 0.86–2.65; *p* = 0.15). When stratified by age, high PNI was also significantly associated with better OS in patients aged 65 years and over (HR = 2.23, 95% CI: 1.71–2.89; *p* <0.001) [18]. Similarly, in our study, a high PNI level was found to be associated with better OS both in the entire cohort and in patients aged 65 years and over [HR = 0.49 (95% CI: 0.26–0.92), *p* = 0.02 and HR = 0.45 (95% CI: 0.24–0.84), *p* = 0.01, respectively]. The current literature evaluating the relationship between the Prognostic Nutritional Index and survival outcomes in lung cancer patients treated with immune checkpoint inhibitors is summarized in Figure 5 [19,20,21,22,23,24].

GNRI was originally developed to assess the nutrition-related risk of mortality in elderly, non-cancer inpatients. GNRI is calculated using serum albumin levels and ideal body weight. High GNRI has been reported to be associated with favorable survival outcomes in various solid tumors, including non-small cell lung cancer [25,26,27]. In the era of immunotherapy, nutritional status may influence immunological competence and the effectiveness of treatment. As immune checkpoint inhibitors become established as a cornerstone in the management of metastatic NSCLC, the identification of reliable and non-invasive prognostic markers such as GNRI is becoming increasingly important.

Several studies have evaluated the prognostic value of GNRI in patients with metastatic non-small-cell lung cancer receiving immune checkpoint inhibitors. In a retrospective cohort study involving 85 patients with advanced NSCLC treated with PD-1 inhibitors, Sonehara et al. demonstrated that a higher GNRI ≥ 89.5 was significantly associated with improved clinical outcomes compared to low GNRI, including prolonged PFS (3.7 vs. 2.4 months; *p* = 0.041) and OS (14.2 vs. 6.1 months; *p* = 0.008). Multivariate analysis further identified high GNRI (≥89.5), ECOG performance status of 0–1, and age of ≥ 70 years as independent prognostic factors for favorable overall survival. Moreover, patients with a high GNRI (≥89.5) were significantly more likely to receive subsequent lines of systemic therapy following immune checkpoint inhibitor treatment compared to those with a low GNRI (<89.5) [(68.6% vs. 33.3%; *p* = 0.008)], highlighting the potential role of nutritional status in sustaining treatment eligibility and improving long-term outcomes in this population [28]. Similarly, Jiang et al. evaluated the prognostic significance of the GNRI in a cohort of 148 patients with NSCLC treated with immune checkpoint inhibitors. Using ROC curve analysis, the optimal GNRI cutoff was identified as 108.15 [area under the curve (AUC) = 0.575, *p* = 0.048]. Multivariate Cox regression analysis demonstrated that a high GNRI was independently associated with improved OS [hazard ratio (HR): 0.536, *p* = 0.03]. Notably, subgroup analyses revealed that the prognostic utility of GNRI was particularly evident in patients with tumor–node–metastasis (TNM) stage II–III disease and those aged ≥ 65 years, highlighting its potential role as a clinically relevant biomarker in selected populations undergoing immunotherapy [29].

A study by Karayama et al. indicated that among 158 patients with NSCLC treated with nivolumab, a low GNRI was correlated with a significantly shorter PFS [median: 1.9 months; 95% CI: 0.6–3.3 months] compared to moderate (median: 4.0 months; 95% CI: 2.3–5.8 months; *p* = 0.017) and high (median: 3.0 months; 95% CI: 1.9–7.2 months; *p* = 0.014) GNRI. A low GNRI was associated with a significantly shorter OS (median: 7.8 months; 95% CI: 2.6–12.0 months) compared to moderate (median: 13.0 months; 95% CI: 9.6–15.2 months; *p* = 0.006) and high (median: 20.6 months; 95% CI: 15.6 months reached; *p* < 0.001) GNRI [30]. Two additional studies also demonstrated that a high GNRI is associated with prolonged OS [31,32]. In the present study, a high GNRI level was associated with improved OS in patients aged 65 years and over [HR = 0.40; 95% CI: 0.24–0.80; *p* = 0.01]. However, GNRI was not identified as an independent prognostic factor in the multivariate analysis.

This study has several limitations that warrant consideration. First, its retrospective, multicenter, single-arm design may have introduced selection bias, limiting the external validity of the findings. Second, data regarding the nutritional status of patients at the time of lung cancer diagnosis were not available, as such information was not routinely documented during the initial diagnostic process. Therefore, our analysis focused on nutritional and inflammatory indices measured at the initiation of second-line nivolumab therapy. Third, the sample size, particularly in certain subgroups such as patients with PD-L1 ≥ 1%, may have reduced the statistical power to detect statistically significant associations. Additionally, the predominance of male patients in the study population (85.2%) likely reflects regional smoking patterns and sex-related epidemiological differences in NSCLC [33]. However, this imbalance may limit the generalizability of the findings to female patients. Lastly, the use of multiple inflammatory and nutritional biomarkers, each with variable cut-off values across studies, introduces a degree of heterogeneity that may affect the reproducibility and clinical interpretation of prognostic associations.

## 5. Conclusions

This study highlighted the prognostic significance of the PNI in patients with NSCLC treated with immune checkpoint inhibitors, with a PNI cut-off of ≥ 45.2 emerging as an independent predictor of overall survival, particularly in elderly patients. These findings support the integration of simple, cost-effective nutritional biomarkers into routine clinical assessment for better risk stratification; notably, the PNI could also guide nutritional interventions prior to the initiation of immunotherapy. Prospective studies are warranted to validate the prognostic value of the PNI in larger and more diverse patient cohorts.

## Figures and Tables

**Table 1:** Baseline demographic and clinicopathologic findings for the whole cohort.

Variables		*n* = 196 (%)
Age (years)	Median (range)	65 (23–88)
	≥65	101 (51.5)
Gender	Female	29 (14.8)
	Male	167 (85.2)
Smoking	Never a smoker	30 (15.3)
	Ex-smoker	114 (58.2)
	Active smoker	52 (26.5)
Smoking duration (pack/years) (median) (range)		40 (10–120)
ECOG PS	PS 0	53 (27)
	PS 1	130 (66.3)
	PS ≥ 2	13 (6.7)
Body mass index (kg/m^2^) (median) (range)		24.4 (14.8–37.04)
Histopathology	Adenocarcinoma	101 (51.5)
	Squamous cell carcinoma	82 (41.8)
	NOS	13 (6.6)
Stage at diagnosis	Stage 1	10 (5.1%)
	Stage 2	35 (17.9%)
	Stage 3	52 (26.5%)
	Stage 4	99 (50.5%)
PD-L1 status	PD-L1 < 1%	67 (34.6)
	PD-L1 ≥ 1%	69 (35.2)
	Unknown	60 (30.6)
PD-L1 level (median %) (range)		35 (1–95)
Prognostic Nutritional Index (PNI) (median) (range)		45.2 (23.0–73.9)
Geriatric Nutritional Index (GNR) (median) (range)		103.8 (64.6–139.31)
Inflammatory Burden Index (IBI) (median) (range)		60.2 (0.17–2362.5)
Neutrophil/Lymphocyte Ratio (NLR) (median) (range)		3.7 (0.9–21.5)
Platelet/Lymphocyte Ratio (PLR) (median) (range)		196.8 (26.6–1970)
Status	Exitus	74 (37.8)
	Alive	122 (62.2)
Overall survival	Median (months)	12.4 (95% CI: 8.4–16.4)
	1 years (%)	53
	2 years (%)	25

ECOG PS: ECOG performance status, NOS: not otherwise specified, PD-L1: programmed death ligand 1.

**Table 2:** Univariate analysis results for overall survival in the whole cohort.

Variables		Median OS (Months) (95% CI)	Univariate Analysis	
			HR (95% CI)	*p*
Age	≥65	12.1 (8.7–15.5)	1.2(0.7–1.9)	0.37
	<65	14.2 (6.5–21.9)		
Gender	Female	9.7 (not calculated)	0.8 (0.4–1.5)	0.55
	Male	14.2 (8.8–19.6)		
BMI	<25	11.9 (8.0–15.8)	0.97 (0.92–1.0)	0.41
	≥25	17.8 (6.1–29.6)		
Smoking	Never a smoker	7.2 (6.1–8.3)	0.6 (0.1–1.1)	0.21
	Active-ex	14.2 (9.1–19.3)		
ECOG PS	PS 0–1	14.2 (8.8–19.6)	4.2 (2.1–8.4)	<0.01
	PS ≥ 2	5.1 (0.84–9.4)		
PD-L1	PD-L1 < 1%	10.6 (6.7–14.5)	1.02 (0.59–1.7)	0.93
	PD-L1 ≥ 1%	12.1 (6.8–17.4)		
	PD-L1 < 50%	10.6 (7.8–13.4)	0.9 (0.5–1.8)	0.90
	PD-L1 ≥ 50%	14.6 (3.5–25.7)		
NLR	<3.3	17.8 (10.6–25.1)	2.5 (1.5–4.2)	<0.01
	≥3.3	8.03 (4.7–11.3)		
PLR	<196.8	17.8 (10.5–25.2)	1.7 (1.1–2.8)	0.01
	≥196.8	9.7 (4.6–14.9)		
PNI	<45.2	7.1 (1.5–12.8)	0.5 (0.3–0.8)	0.004
	≥45.2	17.8 (11.7–24.03)		
IBI	<60.2	11.9 (3.4–20.3)	1.4 (0.8–2.3)	0.13
	≥60.2	12.1 (5.9–18.3)		
GNRI	<98.0	5.1 (2.3–7.8)	0.4 (0.24–0.8)	0.01
	≥98.0	Not reached		

**Table 3:** Univariate analysis results for overall survival in patients aged 65 years and older.

Variables		Median OS (Months) (95% CI)	Univariate Analysis	
			HR (95% CI)	*p*
Gender	Female	Not reached	1.5 (0.5–4.2)	0.43
	Male	12.1 (9.3–14.9)		
BMI	<25	14.6 (8.4–20.7)	1.07 (0.56–2.0)	0.82
	≥25	9.8 (6.6–13.0)		
Smoking	Never a smoker	Not reached	1.1 (0.3–3.7)	0.82
	Active–ex	12.1 (8.6–15.6)		
ECOG PS	PS 0–1	12.4 (9.7–15.7)	3.04 (1.0–8.6)	0.03
	PS ≥ 2	5,1 (1.0–11.4)		
PD-L1	PD-L1 < 1%	10.1 (5.6–14.5)	0.8 (0.4–1.7)	0.67
	PD-L1 ≥ 1%	14.6 (9.1–20.1)		
	PD-L1 < 50%	12.4 (9.1–15.1)	0.4 (0.1–1.5)	0.22
	PD-L1 ≥ 50%	14.6 (1.0–30.9)		
NLR	<3.3	Not reached	1.6 (0.8–3.2)	0.12
	≥3.3	12.1 (4.9–19.3)		
PLR	<196.8	12.1 (9.6–14.6)	1.5 (0.8–2.8)	0.17
	≥196.8	14.6 (0.65–28.6)		
PNI	<45.2	5.2 (0.1–12.1)	0.4 (0.2–1.1)	0.01
	≥45.2	12.4 (9.1–15.7)		
IBI	<60.2	10.1 (6.1–14.08)	1.4 (0.7–2.7)	0.28
	≥60.2	12.1 (5.7–18.5)		
GNRI	<98.0	5.1 (2.3–7.8)	0.4 (0.24–0.8)	0.01
	≥98.0	Not reached		

## Data Availability

The data presented in this study are available upon reasonable request from the corresponding author. The data are not publicly available due to privacy and ethical restrictions.

## Abbreviations

The following abbreviations are used in this manuscript:

BMI	Body Mass Index.
CRP	C-Reactive Protein.
ECOG PS	Eastern Cooperative Oncology Group Performance Status.
GNRI	Geriatric Nutritional Risk Index.
HR	Hazard Ratio.
IBI	Inflammatory Burden Index.
ICIs	Immune Checkpoint Inhibitors.
NLR	Neutrophil-to-Lymphocyte Ratio.
NOS	Not Otherwise Specified
NSCLC	Non-Small-Cell Lung Cancer.
OS	Overall Survival.
PD-1	Programmed Death-1.
PD-L1	Programmed Death Ligand-1.
PFS	Progression-Free Survival.
PLR	Platelet-to-Lymphocyte Ratio.
PNI	Prognostic Nutritional Index.
ROC	Receiver Operating Characteristic.
TNM	Tumor Node Metastasis.
